# Comparison of mid-term clinical outcome in heart transplantation patients using mycophenolate mofetil vs. enteric-coated mycophenolate sodium

**DOI:** 10.3389/fcvm.2022.957299

**Published:** 2022-08-23

**Authors:** Kina Jeon, Darae Kim, Jin-Oh Choi, Yang Hyun Cho, Kiick Sung, Jaewon Oh, Hyun Jai Cho, Sung-Ho Jung, Hae-Young Lee, Jin Joo Park, Dong-Ju Choi, Seok-Min Kang, Jae-Joong Kim, Eun-Seok Jeon

**Affiliations:** ^1^Division of Cardiology, Department of Medicine, Samsung Medical Center, Sungkyunkwan University School of Medicine, Seoul, South Korea; ^2^Department of Thoracic and Cardiovascular Surgery, Samsung Medical Center, Sungkyunkwan University School of Medicine, Seoul, South Korea; ^3^Department of Internal Medicine, Yonsei University College of Medicine, Seoul, South Korea; ^4^Department of Internal Medicine, Seoul National University College of Medicine, Seoul, South Korea; ^5^Department of Thoracic Surgery, Asan Medical Center, University of Ulsan College of Medicine, Seoul, South Korea; ^6^Division of Cardiology, Department of Internal Medicine, Seoul National University Bundang Hospital, Seongnam, South Korea; ^7^Department of Internal Medicine, Asan Medical Center, University of Ulsan College of Medicine, Seoul, South Korea

**Keywords:** heart transplantation, prognosis, mycophenolate mofetil, mycophenolic acid, rejection

## Abstract

**Background:**

Mycophenolate mofetil (MMF) is a prodrug of mycophenolic acid (MPA) and a key immunosuppressant for improving graft survival in patients with heart transplantation (HTx). However, dose reduction or interruption is occasionally needed due to gastrointestinal (GI) side effects. Enteric-coated mycophenolate sodium (EC-MPS) is an alternative form of MPA delivery to improve GI tolerability. In the present study, the efficacy of EC-MPS compared with MMF in HTx patients was investigated.

**Methods:**

In this retrospective study, the Korean Organ Transplant Registry (KOTRY) data were used to analyze the efficacy and rejection rate of MMF and EC-MPS. A total of 611 patients was enrolled from 2014 to February of 2021. Patients were divided based on the use of MMF or EC-MPS at 6 months post-HTx. Patients who were not prescribed MMF or EC-MPS were excluded. Graft survival, all-cause mortality, and treated rejection were compared between the two groups. All statistical analyses were performed using SPSS; characteristics were compared using Pearson chi-square test and survival rate with Kaplan-Meier plot and log-rank test.

**Results:**

A total of 510 HTx patients was analyzed (mean age: 51.74 ± 13.16 years, males: 68.2%). At 6 months after HTx, 78 patients were taking EC-MPA (12.8%) and 432 patients were taking MMF (70.7%). The median follow-up was 42.0 months (IQR: 21.7–61.0 months). Post-HTx outcomes including overall survival, all cause mortality, acute cell mediated rejection (ACR), acute antibody mediated rejection (AMR), treated rejection, and cardiac allograft vasculopathy (CAV) were comparable between the two groups during follow-up.

**Conclusion:**

Notable differences were not observed in overall survival, all cause mortality, ACR, AMR, treated rejection, and CAV between MMF and EC-MPS groups. Efficacy of EC-MPS was similar to that of MMF in HTx patients during mid-term follow up after HTx.

## Introduction

Heart transplantation (HTx) is the standard treatment for end-stage heart failure (HF). Survival and prognosis of HTx have improved over the last two decades with introduction of effective immunosuppression therapy (1, 2). Generally, for maintenance immunosuppression, HTx patients receive a combination of two or three classes of medication, calcineurin inhibitors (CNIs), anti-metabolites, and proliferation signal inhibitors (3).

Mycophenolate mofetil (MMF, CellCept®, Roche Laboratories, Nutley, NJ, USA) is a potent anti-proliferative drug that recently replaced azathioprine as the drug of choice due to improved survival and reduced rejection rates compared with azathioprine (4). MMF is a prodrug of mycophenolic acid (MPA) and inhibits inosine-5′-monophosphate dehydrogenase to block proliferation of T and B cells, leading to repression of both cell- and humoral-mediated immunity (5). However, gastrointestinal (GI) intolerance is a common dose-limiting side effect often leading to interruptions in therapy, which increases risk of rejection (6). Enteric-coated mycophenolate sodium (EC-MPS, Myfortic^®^, Novartis Pharmaceuticals, East Hanover, NJ, USA) was developed to reduce GI effects of MMF. Clinical trials in kidney transplant recipients demonstrated that EC-MPS is therapeutically equivalent to MMF (7, 8).

Unlike kidney transplantations, data regarding long-term HTx outcome with use of EC-MPS are limited. In the present study, using a nationwide organ transplant registry in Korea, post-HTx outcome was evaluated between patients taking MMF or EC-MPS in combination with CNIs and corticosteroids.

## Methods

### Study population

The nationwide multi-center HTx data submitted to the Korean Heart Transplant Registry (KOTRY), the first nationwide organ transplantation registry in Korea, was used in the present study (9). From 2014 to 2019, a total of 611 patients underwent HTx. With the exclusion of follow-up losses, a final number of 510 patients were included in this study. The study was reviewed and approved by the institutional review board of each transplantation center. The KOTRY registry includes baseline and follow-up data of transplanted patients. After HTx, follow-up visits were recorded at 1, 6, and 12 months and annually thereafter. Patients were classified into MMF or EC-MPS groups based on immunosuppressive regimen at 6 months after HTx.

### Immunosuppression

CNI-based triple immunosuppressive therapy (tacrolimus, mycophenolate mofetil, and prednisone) was initially administered as maintenance therapy to most patients. Cyclosporine was administered if patients developed severe side effects from tacrolimus, such as seizures or encephalopathy. A regimen using a mammalian target of rapamycin (mTOR) inhibitor, either sirolimus or everolimus, in place of a CNI-free regimen was prescribed to eligible patients, including those with renal insufficiency or malignancy. An mTOR inhibitor was administered in conjunction with a CNI in patients who developed rejection with graft dysfunction, cytomegalovirus infection, or cardiac allograft vasculopathy (CAV). In case of intolerance to an mTOR inhibitor, a conventional CNI-based regimen was maintained. Patients at low risk of rejection were tapered off steroids 6 months after HTx according to transplantation clinic protocol. All HTx recipients underwent a protocol-based regular evaluation at their transplantation clinic (10). Post-HTx clinical outcome included overall survival, freedom from angiographic CAV (11), and any treated rejection. Rejection was diagnosed through endomyocardial biopsy and included both acute cellular rejection (ACR) and antibody-mediated rejection (AMR). Rejections were defined according to the revised International Society for Heart and Lung Transplantation (ISHLT) classification (12). Treated rejection was defined as events that require either intravenous steroids for acute cellular rejections or rituximab injections for antibody-mediated rejections.

### Statistical analysis

Continuous variables are recorded as mean ± standard deviation, and categorical variables are reported as frequency and percentages. Baseline recipient/donor characteristics and clinical outcomes of HTx were compared between MMF and EC-MPS groups. Post-HTx outcomes included treated rejection and all cause mortality. The two groups were compared using chi-square test, and continuous variables using Student's t-test. The cumulative incidence of events and outcome analysis was assessed using the Kaplan-Meier method, and statistical significance was calculated using the log-rank test. Due to the number difference between the two groups, 1 to 1 individual matching within caliper by propensity score matching was performed. Analysis by chi-square test was used for group comparison, and two sample t-test was used for continuous variables. All data were analyzed using SPSS version 25.0 (SPSS Inc., Chicago, IL, USA) and R-version 4.2.0 (13).

## Results

### Baseline characteristics

Among the 510 HTx patients, 432 were taking MMF (70.7%) and 78 were taking EC-MPS (12.8%) post-HTx. Patients in EC-MPS group were younger and had longer warm ischemic time, aortic-cross clamp, and cardiopulmonary bypass time (Table 1). Significantly more patients in the EC-MPS group received extracorporeal membrane oxygenation before HTx (31.5 vs. 19.2%, *p* = 0.031). In addition, significantly more patients (65.3 vs. 91.0%, *p* < 0.001) in the EC-MPS group remained on steroid treatment at 6 months post-HTx compared with subjects in the MMF group. An average dose of 1,500 mg (1,000–2,000 mg) of MMF and 1,080 mg (720–1,440 mg) of EC-MPS was used in each group. This amount is an equivalent dose of the active form, MPA.

**Table 1:** Comparison of donor/recipient baseline characteristics between the MMF and EC-MPS groups.

	**MMF group** **(n = 432)**	**EC-MPS group** **(n = 78)**	***p*-value**
Age, years			
Recipient	52.4 ± 12.6	48 ± 15.5	0.001
Donor	39.8 ± 11.4	40.4 ± 10.8	0.184
Recipient			
BMI (kg/m^2^)	22.6 ± 3.7	22.4 ± 3.5	0.898
Sex (male)			
Recipient	290 (66.9%)	59 (75.6%)	0.146
Donor	301 (69.7%)	53 (67.9%)	0.790
Male recipient/female donor	64 (14.8%)	24 (16.7%)	0.731
Female recipient/male donor	76 (17.6%)	7 (9.0%)	0.066
Recipient			
Hypertension	109 (25.2%)	40 (51.3%)	0.035
Diabetes mellitus	117 (27.1%)	35 (44.9%)	0.277
Chronic kidney disease	62 (14.4%)	13 (16.7%)	0.055
Previous malignancy	33 (7.6%)	6 (7.7%)	1.000
Cold ischemia time (min)	113.5 ± 59.2	103.6 ± 64.1	0.323
Warm ischemia time (min)	56.9 ± 25.6	75.5 ± 44.7	0.001
ACC time (min)	113.1 ± 49.1	140.9 ± 52.5	<0.001
CPB time (min)	152.3 ± 65.1	181.3 ± 66.5	<0.001
Most recent PRA > 10%			
Overall	171 (39.6%)	33 (42.3%)	0.707
Class I	124 (29.0%)	21 (27.3%)	0.891
Class II	116 (26.9%)	24 (30.8%)	0.492
LVEF at HTx	26.8 ± 15.1	27.0 ± 12.8	0.698
Cr at time of HTx (mg/dL)	1.21 ± 0.87	1.43 ± 1.59	0.070
Diagnosis			
Dilated cardiomyopathy	224 (51.9%)	39 (50.0%)	0.806
Ischemia	89 (20.6%)	13 (16.7%)	0.538
Retransplant	15 (3.5%)	3 (3.8%)	0.750
**Pre-HTx support**			
Mechanical ventilator	11 (14.1%)	102 (23.6%)	0.075
ECMO	136 (31.5%)	15 (19.2%)	0.031
LVAD	21 (4.9%)	4 (5.1%)	1.000
Induction therapy	372 (86.1%)	71 (91.0%)	0.278
**Immunosuppression at 6 months post-HTx**			
Tacrolimus	368 (86.2%)	66 (84.6%)	0.863
Cyclosporine	11 (2.5%)	6 (7.7%)	0.032
Everolimus	111 (25.7%)	23 (29.5%)	0.487
Steroid	282 (65.3%)	71 (91.0%)	<0.001

*Data are shown as mean ± standard deviation or number*.

*ACC, aortic cross-clamp; BMI, body mass index; CPB, cardiopulmonary bypass; Cr, creatinine; ECMO, extracorporeal membrane oxygenation; HLA, human leukocyte antigen; IABP, intraaortic balloon pump; LVAD, left ventricular assisting device; PRA, panel reactive antibodies; RVSP, right ventricular systolic pressure; HTx, heart transplantation; MMF, mycophenolate mofetil; EC-MPS, enteric-coated mycophenolate sodium*.

### Post-HTx clinical outcomes

**Figure 1:**
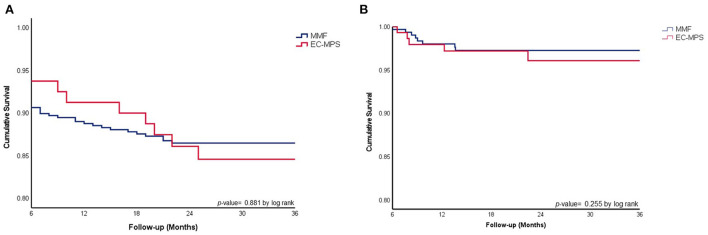
**(A)** Comparison of overall survival between HTx patients in EC-MPS and MMF groups. **(B)** Cumulative survival in freedom from treated rejection in heart transplantation patients using MMF vs. EC-MPS. Overall survival rates **(A)** and cumulative survival in freedom from treated rejection **(B)** were similar between HTx patients in the EC-MPS and MMF groups. HTx, heart transplantation; EC-MPS, enteric-coated mycophenolate sodium; MMF, mycophenolate mofetil.

Significant differences in all-cause mortality and freedom from treated rejection were not observed between the two groups (Figures 1A,B) during the mean follow-up period (40 ± 23 months). Post-Hx overall survival, all cause mortality, freedom from ACR, AMR, and CAV were similar between the two groups (Table 2). Although significantly more patients in the EC-MPS group (4.4 vs. 10.3%, *p* = 0.049) experienced treated rejection during the first year compared with the MMF group, graft survival and overall survival were comparable between two groups. During follow-up, incidences of an infection requiring hospitalization were similar between the two groups.

**Table 2:** Comparison of clinical outcomes after HTx between the MMF and EC-MPS groups.

	**MMF group** **(n = 432)**	**EC-MPS group** **(n = 78)**	***p*-value**
All cause mortality	61 (14.1%)	12 (15.4%)	0.855
1-year freedom from ACR	195 (45.1%)	29 (37.2%)	0.325
3-year freedom from ACR	180 (41.7%)	24 (30.7%)	0.567
1-year freedom from AMR	416 (96.3%)	72 (92.3%)	0.942
3-year freedom from AMR	415 (96.3%)	72 (92.3%)	0.567
1-year freedom from treated rejection	414 (95.8%)	71 (91.0%)	0.047
3-year freedom from treated rejection	408 (95.8%)	68 (87.2%)	0.658
1-year freedom from CAV	418 (96.7%)	72 (92.3%)	0.078
3-year freedom from CAV	390 (90.3%)	67 (85.9%)	0.490
Infection requiring hospitalization	15 (3.5%)	3 (3.8)	0.746

*HTx, heart transplantation; MMF, mycophenolate mofetil; EC-MPS, enteric-coated mycophenolate sodium; ACR, Acute cell mediated rejection; AMR, Acute antibody mediated rejection; CAV, cardiac allograft vasculopathy*.

We performed a subgroup analysis of patients treated with cyclosporine, because cyclosporine is known to influence MPA pharmacokinetics. Post-HTx clinical outcomes were comparable between MMF and EC-MPS group in subgroup of patients who were treated with cyclosporine (Supplementary Table 1).

Due to significant difference of baseline characteristics between MMF and EC-MPS groups, a propensity score matching analysis was conducted with adjustment of age, gender, pre-HTx ECMO and prolonged steroid use (Table 3). In this analysis, post-HTx clinical outcomes including all-cause mortality were comparable between two groups.

**Table 3:** 1 to 1 individual matching between MMF vs. EC-MPS within caliper by propensity score.

	**MMF group**	**EC-MPS group**	***p*-value**
Number	75	75	
Sex (male)	55.0 (73.3%)	58.0 (77.3%)	0.41
Age (years)	46.39 ± 14.90	47.53 ± 15.33	0.341
Pre-HTx support, ECMO	19.0 ± 25.3	21.0 ± 28.0	0.62
Steroid use at 6 months	74.0 (98.7%)	74.0 (98.7%)	1
All cause mortality	11 (14.7%)	12 (16%)	0.7
1-year freedom from ACR	27 (36.0%)	31 (41.3%)	0.346
3-year freedom from ACR	25 (33.3%)	26 (34.7%)	0.168
1-year freedom from AMR	72 (96.0%)	72 (96.0%)	0.914
3-year freedom from AMR	71 (94.7%)	72 (96.0%)	0.168
1-year freedom from treated rejection	27 (36.0%)	23 (30.7%)	0.488
3-year freedom from treated rejection	21 (28.0%)	20 (26.7%)	0.855
1-year freedom from CAV	70 (93.3%)	71 (94.7%)	0.754
3-year freedom from CAV	68 (90.7%)	68 (90.7%)	1

*HTx, heart transplantation; MMF, mycophenolate mofetil; EC-MPS, enteric-coated mycophenolate sodium; ACR, Acute cell mediated rejection; AMR, Acute antibody mediated rejection; CAV, cardiac allograft vasculopathy*.

## Discussion

To the best of our knowledge, this is the first mid-term study in which post-HTx outcomes from a multi-center registry were compared between patients receiving MMF or EC-MPS. During the mean follow-up of 40 months, post-HTx clinical outcomes were similar between MMF and EC-MPS groups, although significantly more patients with EC-MPS experienced treated rejection at 1-year follow-up.

MMF, due to its effectiveness in reducing acute rejection rates (4), is now the drug of choice for post-transplantation immunosuppression in multiple organs including the heart. However, GI side effects caused by MPA, the active form of MMF (14, 15), and leukopenia are dose-limiting side effects. Dose reduction due to MMF intolerance increases the risk of acute rejection (16). In previous kidney transplantation registries, the incidence of MMF intolerance leading to dose reductions reportedly ranged from 42 to 59% (15–17). In a single-center study, the recommended dose of 3 mg/day of MMF in adult HTx patients was poorly tolerated, and the median dose of MMF at 6 months post-HTx was 1,560 ± 984 mg/day (18), similar to median doses of MMF at 6 months post-HTx in the present study. In the study cohort, 12.8% were taking EC-MPS at 6 months after HTx because patients either experienced or were predisposed to GI disorder.

EC-MPS was developed to decelerate the release of MPA, contrary to MMF, which shows instant release of MPA into the GI tract. In pharmacokinetic studies, despite delayed delivery, administration of EC-MPS resulted in a similar maximal plasma concentration and MPA exposure (19, 20). In maintenance and *de novo* trials, similar safety and efficacy of EC-MPS compared with MMF were observed in kidney transplant patients with similar rejection, infection, and graft survival at 12 months post-kidney transplant (7, 8).

In a previous single-blinded multi-center trial including 154 HTx patients, similar rates of treatment failure and combination of treated acute rejection, graft loss, and death at 6 months (52.6 vs. 57.9%) and 12 months (57.7 vs. 60.5%) (21, 22) post-HTx were reported. A previous single center randomized study showed that EC-MPS treated HTx patients are less likely to require multiple dose reductions than those on MMF and was associated with a significant lower incidence of treated rejection (23). However, these studies are limited due to a short follow-up of 12 months with a small numbers of patients. In the present study, we analyzed real-world clinical outcomes after HTx with nation-wide multi-center data and longer follow-up duration. Results of the present study indicate similar long-term efficacy of EC-MPS to MMF in HTx patients compared to previous studies (20–22). In addition, our study showed that eventually the average of MPA dose in both groups did not significantly differ.

## Limitations

The present study had limitations due to the retrospective design and the different number of subjects in the MMF and EC-MPS groups. Data regarding MPA level were lacking, as this is not a routine practice in many centers. Time-dependent dose change of MMF and EC-MPS might not have been reported due to pre-specified follow-up intervals in the KOTRY registry and other immunosuppressive regimens determined based on the protocol of each transplantation clinic.

The main use of EC-MPS being an alternative for MMF intolerance due to its gastrointestinal side effects, and this study might been more profound if the actual incidence and severity of GI side effects was assessed after HTx. Unfortunately, due to the nature of our retrospective study, the severity and rate of intolerance were not consistently assessed with objective tools in this registry, therefore, difference in gastrointestinal complications between two groups could not be provided. However, the present study is valuable because our study described and compared real world post-HTx clinical outcomes between MMF and EC-MPS groups from a multi-center, nationwide registry.

## Conclusion

In conclusion, HTx patients treated with EC-MPS and MMF have similar incidence of overall survival, ACR, AMR, treated rejection, CAV, and all cause mortality. Mid-term post-HTx clinical efficacy of EC-MPS was similar to that of MMF in HTx patients.

## Data availability statement

Relevant data are available from the corresponding author on reasonable request.

## Ethics statement

The studies involving human participants were reviewed and approved by KOTRY. The patients/participants provided their written informed consent to participate in this study.

## Author contributions

All authors listed have made a substantial, direct, and intellectual contribution to the work and approved it for publication.

## Funding

This research was supported by a fund (2014-ER6301-00, 2014-ER6301-01, 2014-ER6301-02, 2017-ER6301-00, 2017-ER6301-01, and 2017-ER6301-02) by Research of Korea Centers for Disease Control and Prevention Agency. The funders had no role in study design, data collection and analysis, decision to publish, or preparation of the manuscript.

## Conflict of interest

The authors declare that the research was conducted in the absence of any commercial or financial relationships that could be construed as a potential conflict of interest.

## Publisher's note

All claims expressed in this article are solely those of the authors and do not necessarily represent those of their affiliated organizations, or those of the publisher, the editors and the reviewers. Any product that may be evaluated in this article, or claim that may be made by its manufacturer, is not guaranteed or endorsed by the publisher.
