# Supplementary material for: Comparison of mid-term clinical outcome in heart transplantation patients using mycophenolate mofetil vs. enteric-coated mycophenolate sodium
Source: Front Cardiovasc Med. 2022 Aug 23;9:957299. doi: 10.3389/fcvm.2022.957299 (PMC9448250; doi:10.3389/fcvm.2022.957299)
Supplement: Supplementary file 1 [file Table_1.docx]

**Supplementary Table 1.** Subgroup analysis of all-cause mortality after HTx between the MMF and EC-MPS groups with cyclosporin use

|  |  | MMF group  (n = 432) | EC-MPS group  (n = 78) | p-value |
| --- | --- | --- | --- | --- |
| All-cause mortality | With cyclosporin | 7 (1.6%) | 8 (10.3%) | 0.266 |
|  | Without cyclosporin | 48 (11.1%)8/ | 11 (14.1%) |  |

HTx = heart transplantation; MMF = mycophenolate mofetil; EC-MPS = enteric-coated mycophenolate sodium
